# Cryptococcal Antigen Screening in Asymptomatic HIV-Infected Antiretroviral Naïve Patients in Cameroon and Evaluation of the New Semi-Quantitative Biosynex CryptoPS Test

**DOI:** 10.3389/fmicb.2018.00409

**Published:** 2018-03-13

**Authors:** Elvis Temfack, Charles Kouanfack, Leonella Mossiang, Angela Loyse, Marie C. Fonkoua, Síle F. Molloy, Sinata Koulla-Shiro, Eric Delaporte, Françoise Dromer, Thomas Harrison, Olivier Lortholary

**Affiliations:** ^1^Institut Pasteur, Centre National de la Recherche Scientifique, Unité de Mycologie Moléculaire, UMR 2000, Paris, France; ^2^Department of Internal Medicine, Douala General Hospital, Douala, Cameroon; ^3^Day Hospital, Yaoundé Central Hospital, Yaoundé, Cameroon; ^4^Centre for Global Health, Institute for Infection and Immunity, St George’s, University of London, London, United Kingdom; ^5^Microbiology Unit, Centre Pasteur du Cameroun, Yaoundé, Cameroon; ^6^Cameroon Site, French National Agency for Research on AIDS and Viral Hepatitis, Yaounde, Cameroun; ^7^INSERM U1175-IRD UMI 233, University of Montpellier, Montpellier, France; ^8^Infectious Diseases and Tropical Medicine Department, Necker Pasteur Infectious Diseases Centre, Necker-Enfants Malades Hospital, APHP, Institut Hospitalo-Universitaire Imagine, Paris Descartes University, Paris, France

**Keywords:** cryptococcosis, cryptococcal antigen, screening, point of care, lateral flow assay

## Abstract

**Background:** Cryptococcal meningitis (CM) is a major cause of AIDS-related mortality in Africa. Detection of serum cryptococcal antigen (CrAg) predicts development of CM in antiretroviral (ART) naïve HIV-infected patients with severe immune depression. Systematic pre-ART CrAg screening and pre-emptive oral fluconazole is thus recommended. We postulated that a semi-quantitative CrAg screening approach could offer clinically relevant advantages.

**Methods:** ART-naïve asymptomatic adult outpatients with <100 CD_4_ cells/mm^3^ presenting to the Yaoundé Central Hospital, Cameroon were screened for CrAg using the IMMY lateral flow assay (LFA). CrAg positive patients were consented for lumbar puncture and those with proven CM were treated with combination antifungal therapy and those with no CM were offered long-term oral fluconazole. Simultaneous on-site evaluation of CrAg detection using the new LFA Biosynex^®^ CryptoPS test was performed and both tests were subsequently compared to a reference commercialized CrAg enzyme immunoassay (EIA).

**Results:** Prevalence of serum CrAg in 186 screened adults was 7.5% (95%CI: 4.5–12.4). In CrAg positive patients, CM prevalence was 45.5% (95%CI: 18.3–75.7). IMMY and Biosynex CryptoPS strongly agreed in serum, plasma, and cerebrospinal fluid (Kappa: 98.4, 99.5, 100%, respectively, p < 0.001), and disagreed in urine (29 isolated positive CrAg in urine with IMMY, none with Biosynex and none of whom had proven CM). Compared to EIA, serum specificities were 96.6 and 98.3%, respectively. With Biosynex CryptoPS, all CM patients were serum T2-band positive compared to nonewithout CM. Median EIA reciprocal titre was 160 (IQR: 13.5–718.8) and titres >160 strongly correlated with proven CM and Biosynex CryptoPS T2-band positivity. During the 1-year follow up period, there was no incident case of CM among screened patients and overall incidence of all-cause mortality was 31.5 per 100 person-years-at-risk (95%CI: 23.0–43.1).

**Conclusion:** HIV-associated asymptomatic cryptococcosis is common in Cameroon, warranting integrated systematic screening and treatment. Biosynex CryptoPS holds promise, at point of care, for rapidly stratifying CrAg positive patients for optimal management including lumbar puncture and combination antifungal therapy when needed.

**Summary findings:** Prevalence of CrAg and meningitis (CM) is high in Cameroon. Biosynex CryptoPS is comparable to IMMY LFA in CrAg screening. Its T2-band correlates with high antigen titres and CM, thus promising for identifying patients requiring effective induction therapy.

**Note:** This study was presented in part at the 10th International Conference on Cryptococcus and Cryptococcosis (ICCC) in Iguazu in Brazil from 26 to 30^th^ March 2017 and won a prize oral presentation.

## Introduction

Cryptococcal meningitis (CM) is a major cause of morbidity and mortality among patients with advanced HIV infection especially in resource limited settings. Cryptococcosis is responsible for up to 15% of AIDS related mortality of which 75% is in Sub-Saharan Africa (Rajasingham et al., 2017). Global estimates suggest that CM represents the leading cause of adult meningitis worldwide and the second cause of death after tuberculosis (French et al., 2002; Liechty et al., 2007). Though existing evidence of an association between antiretroviral therapy (ART) program implementation and decreased CM incidence (Mirza et al., 2003; Dromer et al., 2004), in spite of efforts to scale up ART coverage, at least 20% of patients still present to ART care with <100 CD_4_ cells/ml (Siedner et al., 2015). More so, in the African setting (Rajasingham et al., 2017), in the latter patients with detectable untreated blood cryptococcal antigen (CrAg), about 25% subsequently develop CM within the first year of ART compared to as few as no cases in CrAg negatives (Jarvis et al., 2009).

In this setting, two strategies to reduce the incidence of CM have been suggested. Universal fluconazole-based primary prophylaxis, which resulted in decreased CM incidence, but had no impact on overall mortality and thus not widely adopted (Chang et al., 2005; Parkes-Ratanshi et al., 2011). The second strategy consisted of pre-ART systematic screening for CrAg in blood in profoundly immune depressed patients followed by pre-emptive administration of fluconazole to CrAg positive patients (Micol et al., 2007; Jarvis et al., 2012). The latter was endorsed by 2011 World Health Organization (WHO) rapid advice (World Health Organization, 2011) which recommended screening either with latex agglutination (LA) test or the Food and Drug Administration (FDA)-approved lateral flow assay (LFA) point of care (POC) immunochromatographic test (IMMY^®^ diagnostics, Norman, OK, United States) (Rajasingham et al., 2012; IMMY, 2017). This approach which has shown survival benefits when coupled with enhanced adherence to ART (Meya et al., 2010; Letang et al., 2015; Mfinanga et al., 2015; Rajasingham et al., 2017), is cost-effective (Meya et al., 2010; Micol et al., 2010; Jarvis et al., 2013; Smith et al., 2013) and has been incorporated into national guidelines of many countries in the Southern part of Africa (Rajasingham et al., 2017). Nevertheless, Central Africa lags in adopting this strategy. Consequently, through this prospective cohort study in Cameroon, we aimed to determine the prevalence of CrAg in blood and asymptomatic CM in ambulatory ART-naïve adults presenting with <100 CD_4_ cells/ml using IMMY LFA and the incidence of CM during first year of ART in the context of screening. A second objective was to assess the performances of a recently developed semi-quantitative immunochromatographic POC test (Biosynex CryptoPS, BIOSYNEX^®^ diagnostics, Strasbourg, France), capable of identifying participants with high CrAg titres, a surrogate of prevalent CM. We ultimately compared the performances of both POC tests to those of the commercialized enzyme immunoassay (EIA), the Premier^®^ CrAg (Meridian Bioscience, Inc., Newtown, OH, United States), considered in here as the reference standard.

## Materials and Methods

### Study Setting

This study was carried out at the Day Hospital of the Yaoundé Central Hospital in Cameroon where the French National Agency for Research on AIDS and Viral Hepatitis (ANRS) center is located. This Day Hospital is a major HIV treatment center which follows up over 9,000 patients presently on ART using updated national guidelines following WHO recommendations (World Health Organization, 2016).

### Study Procedure

This study was carried out in accordance with the recommendations of the Cameroon National Ethics Committee for Research in Human Health with written informed consent from all included subjects. All subjects gave written informed consent in accordance with the Declaration of Helsinki. The protocol was approved by the Cameroon National Ethics Committee for Research in Human Health and Administrative Authorization for research was also obtained from the Cameroon Ministry of Public Health. HIV-infected, ART naïve ambulatory adults (>18 years) presenting with <100 CD_4_ cells/ml between July 2015 and March 2017 with no history of cryptococcal meningitis, were included. A standardized questionnaire was used to anonymously record baseline sociodemographic data as well as current symptoms at each visit.

**FIGURE 1:**
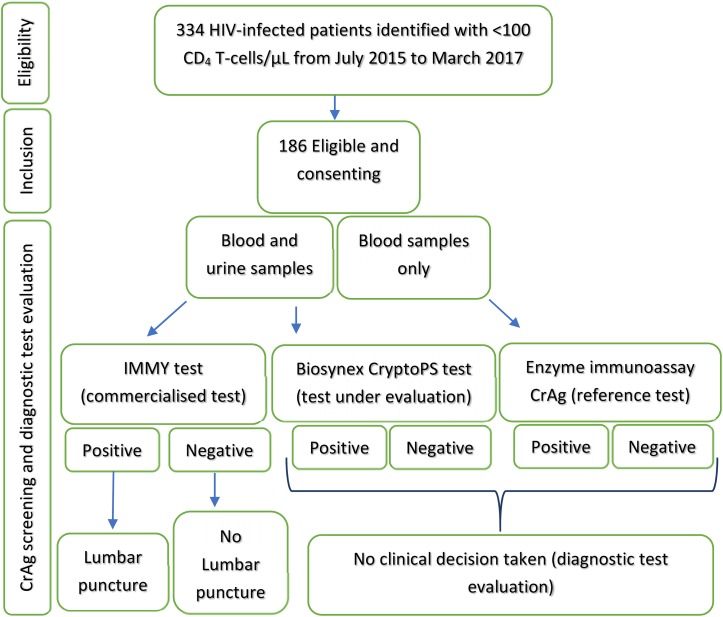
Study procedure: identification, inclusion and screening of participants.

On inclusion, participants were checked for symptoms evocative of CM, and those with recent history of altered mental status or febrile seizures were excluded. In the absence of symptoms, specimens (blood and urine) were collected for same day CrAg screening. CrAg screening was performed in all patients on fresh serum and urine samples using the IMMY LFA (IMMY, 2017) according to manufacturer’s procedure. In addition, all samples were tested and interpreted using a newly developed LFA-based immunochromatographic test called Biosynex CryptoPS without prior knowledge of the results obtained with the IMMY LFA test (**Figure 1**). Additionally, plasma samples obtained on the same day as the serum and urine were also screened with both POC.

Briefly, Biosynex CryptoPS can detect the four serotypes of *Cryptococcus* spp., and provides results within 10 min. This test has two bands: the T1-band is qualitative and the T2-band is semi-quantitative. The T2 band will only appear in patients with elevated CrAg titres (Sriruttan et al., 2016).

Patients were considered CrAg positive if serum samples were positive using the IMMY LFA test but all patients with detectable CrAg in any fluid were consented for lumbar puncture (LP). Freshly obtained cerebrospinal fluid (CSF) was tested with both POC. Direct examination was performed (Indian ink staining) and CSF was cultured on Sabouraud dextrose. Participants with laboratory proven CM (positive India ink and/or culture) were offered treatment within the phase III “Advancing Cryptococcal Meningitis Treatment in Africa” (ACTA) trial (Advancing Cryptococcal Meningitis Treatment for Africa [ACTA], 2013).

Participants with detectable CrAg but no laboratory proven CM were placed on pre-emptive oral fluconazole: 800 mg/day for 2 weeks, then 400 mg/day for 8 weeks and 200 mg/day from week 10 till two consecutive control CD_4_ cell counts at month 6 and 12 were both above 200 cells/ml (World Health Organization, 2011). CrAg negative participants were immediately started on ART without fluconazole, while those on fluconazole and those treated for CM, had ART deferred by 2 and 4 weeks, respectively.

Follow up visits were every 2 weeks for the first 10 weeks, then every 3 months for 1 year. At each visit, symptoms of CM were carefully searched and if clinical suspicion, LP was performed. ART adherence evaluation and support through counseling and medication dispensation were also done. In case of missed appointments, participants or closed family members were followed-up by phone and verbal autopsies conducted for participants who died.

To assess the performances of the LFA tests, we chose the EIA Premier^®^ CrAg test (Meridian Bioscience, Inc., Newtown, OH, United States) as reference standard. Aliquots of the 186 serum samples stored at -80°C at the Centre Pasteur du Cameroun were subsequently transported frozen under rigorous conditions to France for CrAg testing using EIA Premier^®^ according to manufacturer’s procedure. For all EIA CrAg positive specimens, CrAg titre determination was performed by EIA and with both POC test (with Biosynex CryptoPS, both T1 and T2 titres were determined). Results were expressed as reciprocal titre.

### Statistical Analysis

Data were analyzed using STATA 13.0 software (StataCorp, College Station, TX, United States). Results of CrAg screening in the population were expressed as percentages (with their 95% confidence interval [CI]). Categorical variables were compared using Pearson’s Chi-square or Fischer’s exact test as required. Continuous variables were reported as means (and standard deviation [SD]) or median (and interquartile range [IQR]) as required. Student *t*-test or Wilcoxon rank sum test were, respectively, used to compare means or medians as required.

Agreement between IMMY and Biosynex CryptoPS tests in classifying study participants as positive or negative in each specimen type with Indian ink staining or culture of CSF were appraised using Kappa statistics and reported as percentages.

With reference to EIA, two-by-two tables were used to estimate sensitivity and specificity of both POC and reported as percentages with their 95%CI.

Cox proportional hazard ratios (HR) and Kaplan–Meier curves were used to compare survival differences during the first year following CrAg screening. In the multivariate analysis, mortality-associated factors with *p*-value <0.1 in the univariate analysis were included in the final model and reported as adjusted hazard ratios (aHR) with their 95%CI. Evidence against the null hypothesis was considered for a two-tailed *p*-value of <0.05.

## Results

### Prevalence of Latent Cryptococcosis and CM Based on Screening Using IMMY LFA

Between July 2015 and March 2017, 186 patients were screened for CrAg. Mean age was 38.2 years (SD: 10.0) and 67.7% (126/186) were women. Median body mass index was 21.0 kg/m^2^ (IQR: 18.8–23.2) and median CD_4_ T-cells was 44 cells/ml (IQR: 27–75) with no difference between those CrAg positive and negative (**Table 1**).

**Table 1:** Baseline characteristics of study population (*n* = 186) categorized by serum cryptococcal antigen (CrAg) status using IMMY LFA.

Variables at baseline	CrAg positive (*n* = 14)	CrAg negative (*n* = 172)	*p*-value
**Socio-demographic**			
Age in years, mean (SD)	39.3 (10.5)	38.5 (10.2)	0.55
Male, *n* (%)	5 (35.7)	55 (32)	0.77
**Clinical**			
History of tuberculosis, *n* (%)	0	9 (5.3)	0.99
History of liver disease, *n* (%)	0	5 (2.9)	0.99
Body mass index in	20.6	21.0	0.36
Kgm^2^, median (IQR)	(18.4–21.3)	(18.8–23.4)	
**Laboratory**			
CD_4_ cells/ml, median (IQR)	44 (26–76)	48 (35–67)	0.48
Haemoglobin in g/dL, median (IQR)	10.5 (9.6–11.3)	9.9 (8.9–11.3)	0.96
White blood cells × 10^3^/ml, median (IQR)	2 (2.0–5.0)	1 (1–3)	0.05
Alanine transaminase in IU/L, median (IQR)	32.4 (23.7–41.1)	27.6 (19.2–43.3)	0.96
Serum creatinine in g/dL, median (IQR)	1 (0.9–1.2)	1 (0.8–1.2)	0.95

**FIGURE 2:**
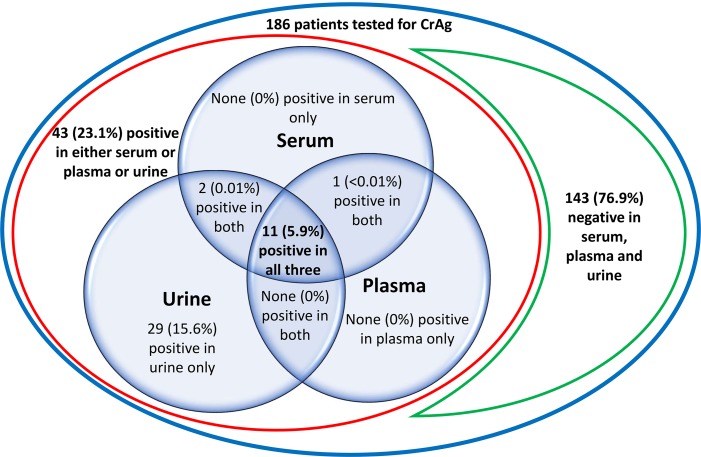
Venn diagram showing the prevalence of cryptococcal antigen (CrAg) in serum, plasma and urine with the IMMY LFA test in 186 HIV-infected asymptomatic antiretroviral naïve severely immune depressed patients.

Prevalence of serum CrAg was 7.5% (14/186) (**Table 2**). Considering all screened specimens (serum/plasma, and urine), 23.1% (43/186) had detectable CrAg (**Figure 2**). Among the 43 patients with detectable CrAg in any site, 27 (62.8%) accepted a LP including 11 of the 14 patients with positive serum CrAg. Four of the 27 LPs did not yield CSF (4 patients with CrAg detection positive in urine only) and 21/23 CSF (91.3%) were cultured (1 positive Indian ink CSF and one isolated urine CrAg were not cultured).

**Table 2:** On site comparison of IMMY diagnostics lateral flow assay (LFA) and Biosynex CryptoPS tests positivity in 186 fresh serum, plasma and urine samples, and 23 cerebrospinal fluid (CSF) samples.

Specimen types	IMMY LFA test % (95% CI)	Biosynex CryptoPS % (95%CI)	% agreement (Kappa)	*p*-value
Serum	7.5	5.9	98.4	0.001
*N* = 186	(4.5–12.4)	(3.3–10.4)		
Plasma	6.5	5.9	99.5	0.001
*N* = 186	(3.7–11.1)	(3.3–10.4)		
Urine	22.6	5.9	83.3	0.001
*N* = 186	(17.1–29.2)	(3.3–10.4)		
CSF	17.4	17.4	100	0.001
*N* = 23	(6.2–40.3)	(6.2–40.3)		

Overall, CM was diagnosed in 21.7% (5/23) of patients by means of a positive Indian ink staining (5/5), positive detection of CrAg (4/5), and positive culture (4/4) (**Table 3**). Clinically, patients with positive serum CrAg detection and CM were not different from those without CM (**Table 3**). Moreover, 3/5 had no signs suggestive of meningitis. None of the 29 patients with isolated positive urine CrAg had CM.

**Table 3:** Baseline clinical and laboratory characteristics of patients screened serum CrAg positive using IMMY LFA and stratified by presence or absence of cryptococcal meningitis (CM).

Variables	With CM (*n* = 5)	Without CM (*n* = 18)	*p*-value
**Symptoms**			
Headache, *n* (%)	3 (60)	2 (11.8)	0.06
Fever, *n* (%)	1 (20)	3 (16.7)	0.99
Confusion, *n* (%)	1 (20)	2 (11.1)	0.54
Photophobia, *n* (%)	1 (20)	1 (5.6)	0.40
**Clinical signs**			
Neck stiffness, *n* (%)	2 (40)	0	0.04
Kernig sign, *n* (%)	2 (40)	0	0.04
Brudzinski sign, *n* (%)	2 (40)	1 (5.6)	0.10
**CSF findings at inclusion**			
IMMY LFA CrAg positive, *n* (%)	4 (80)	0	0.001
Indian ink positive, *n* (%)	5 (100)	0	0.001
Culture positive, *n* (%)	4 (80)	0	0.001
Glucose in g/L, median (IQR)	0.4	0.5	0.08
	(0.4–0.5)	(0.40–0.5)	
Proteins in g/L, median (IQR)	0.5	0.5	0.72
	0.4–0.8)	(0.3–0.7)	

### Subsequent Follow-Up and Outcome in the Study Population

All 5 patients with CM were enrolled in the ACTA trial and all the others with detectable CrAg by IMMY LFA considered as asymptomatic cryptococcosis, were prescribed fluconazole. Median follow-up for the study population was 323 days (IQR: 92–363), not different in serum CrAg positive and negative patients. Median ART initiation was 3 days (IQR: 1–7) in CrAg negative patients and 97.5% of all patients started first line regimen containing tenofovir-lamivudine-efavirenz. Overall adherence to ART and fluconazole was 100%. At 6 and 12 months, median CD_4_ cell/ml were, respectively, 208 (IQR: 129–284) and 256 (IQR: 183–315), similar in CrAg positive and negative patients (*p* = 0.59). There was no incident case of cryptococcal meningitis during the total 123.9 person-years-at-risk (PYAR) follow-up time.

**FIGURE 3:**
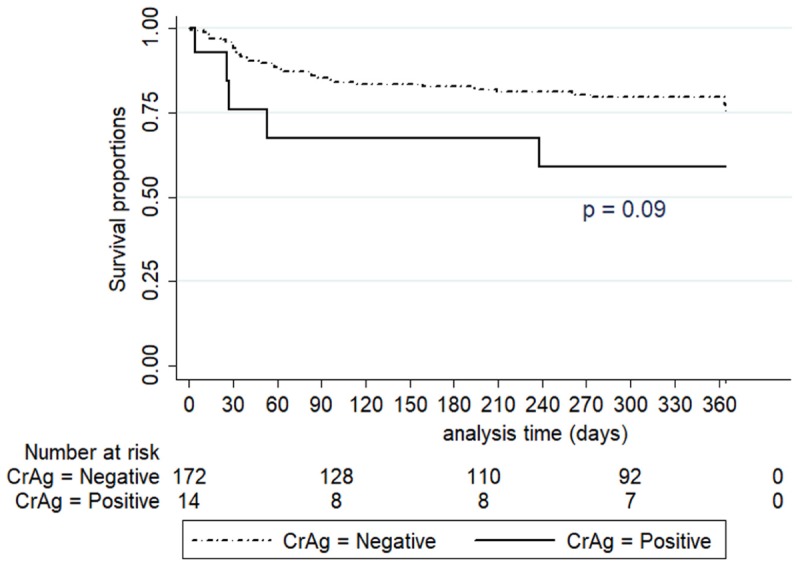
Kaplan–Meier survival estimates by serum CrAg status during first year of follow-up.

Overall, all-cause mortality was 21% (39/186) at a cumulative incidence rate of 31.5 per 100 PYAR, occurring within a median follow-up period of 82 days (IQR: 33–194), 71.8% (28/39) within the first 3 months of ART. The presence of symptoms of respiratory and gastrointestinal infections during follow-up was significantly associated with increased mortality and remained strongly associated with death after adjusting for other factors in the multivariate analysis while baseline CrAg positivity was not (**Table 4**). Serum CrAg positive patients were less likely to survive than those who were CrAg negative, *p* = 0.09 (**Figure 3**). In those with baseline confirmed CM, mortality in the first year was 60% (3/5), with one death attributed to CM (**Table 5**) on day 4 of treatment.

**Table 4:** Factors associated with mortality during the first year of antiretroviral (ART) in patients screened for CrAg.

Factors	Univariable analysis			Multivariable analysis		
	HR	95% CI	*p*-value	aHR	95% CI	*p*-value
Presence of fever	4.2	1.5–12.1	0.007	3.8	1.3–11.2	0.02
Presence of respiratory symptoms	5.2	2.5–10.8	0.001	2.6	1.0–6.3	0.04
Presence of gastrointestinal symptoms	5.4	2.6–11.1	0.001	4.2	1.8–9.7	0.001
Baseline serum CrAg positive (IMMY Diagnostics)	2.2	0.9–5.7	0.09	1.7	0.5–6.2	0.4

**Table 5:** Probable causes of death in 39 patients during first year of ART following CrAg screening.

Probable causes of death	CrAg positive	CrAg negative	Total deaths, *n* (%)
Gastroenteritis	0	12	12 (30.8)
Respiratory/Tuberculosis	0	8	8 (20.5)
Unknown causes	2	6	8 (20.5)
Severe anemia	0	3	3 (7.7)
Kaposi sarcoma	0	2	2 (5.2)
Cryptococcal meningitis	1	0	1 (2.6)
Cerebral toxoplasmosis	1	0	1 (2.6)
Lymphoma	0	1	1 (2.6)
Pulmonary embolism	1	0	1 (2.6)
Severe sepsis	0	1	1 (2.6)
Other meningitis	0	1	1 (2.6)

### Impact of the Test and the Specimen Used for CrAg Screening

With Biosynex CryptoPS, looking at the T1-band results, 5.9% of patients (11/186) were positive in serum (corresponding to 11/14 of the serum positive with IMMY LFA), 5.9% in urine (11/186, corresponding to 11/42 of the urine positive with IMMY LFA), and 17.4% (4/23) in CSF (4/4 of those positive with IMMY LFA) (**Table 2**). No patient was detected with isolated urine CrAg. Of the 11 patients with the positive T1-band in serum, 10 were CrAg positive in urine, and 45.5% (5/11) were also T2-band positive. All 5 patients had CM while none with negative T2-band had. Of note, 2 of the 5 with CM were T2-band positive in urine.

Concerning the classification of specimens as positive or negative, IMMY LFA and Biosynex CryptoPS agreed in 98.4% of serum and 100% of CSF (**Table 2**) but with the IMMY test 15.6% (29/186) of patients who had no detectable CrAg in plasma and serum were CrAg positive in urine (**Figure 2**). These patients were thus considered as having false positive detection of CrAg in urine by the IMMY test. Each POC test had a 98.9% agreement in classifying plasma and serum specimens from the same patient as positive or negative (data not shown).

The 186 stored sera tested with EIA, 4.3% (8/186) were positive. No serum previously categorized as negative by both POC test was found positive by EIA, including those with isolated urine CrAg by IMMY LFA. Thus, considering EIA as reference, IMMY LFA and Biosynex CryptoPS both had a sensitivity of 100% and respective specificities of 96.6% (95%CI: 92.8–98.6) and 98.3% (95%CI: 95.2–99.7).

**FIGURE 4:**
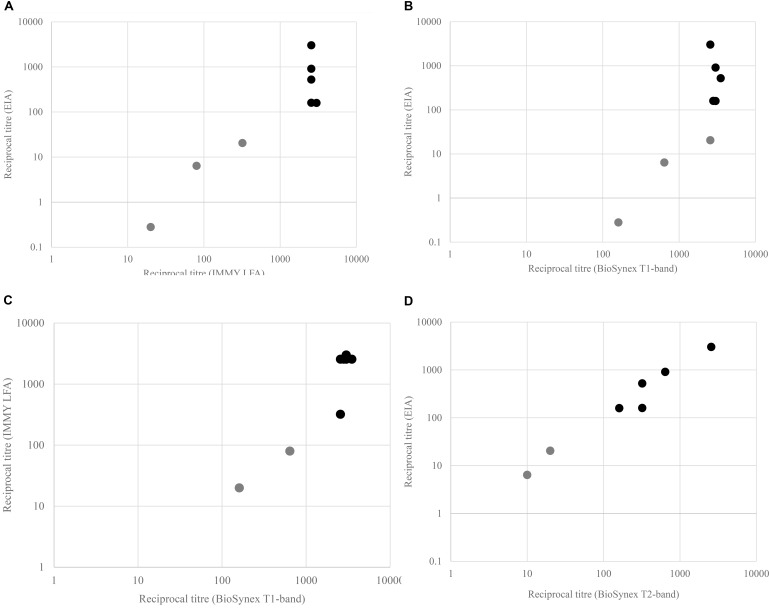
Scatter plots with highlighted points of agreement of CrAg test in classifying patients with elevated serum CrAg. **(A)** Enzyme immunoassay (EIA) CrAg vs. IMMY LFA, **(B)** EIA CrAg vs. Biosynex T1-band, **(C)** IMMY LFA vs. Biosynex T1-band, and **(D)** EIA CrAg vs. Biosynex T2-band.

We then assessed the titres obtained with the three methods. Median EIA CrAg reciprocal titre in serum was 160 (IQR: 13.5–718.8), higher in those with microbiologically confirmed CM: 525 (IQR: 160.5–912.5) than in those without: 6.4 (IQR: 1–20.5), *p* = 0.003. EIA reciprocal titre threshold of 160 was associated with the presence of confirmed CM (*p* = 0.03). Both POC tests CrAg titres agreed with EIA titres in stratifying patients with elevated titres but the T2-band of the Biosynex CryptoPS showed a stronger pattern of agreement (**Figure 4**). CrAg titres were also determined for the 6 serum samples tested negative by EIA, and positive with LFA IMMY (*n* = 6) or Biosynex CryptoPS (*n* = 3). The reciprocal titres ranged between 10 and 20 for those only positive with IMMY, and were at 10 and 10, 10 and >20, 80 and >20 when determined by Biosynex T1-band and IMMY for those positive with both POC.

Considering serum CrAg positivity as a surrogate marker to confirmatory CM diagnosis, the IMMY LFA predicted 45.5% (5/11) of asymptomatic CM while the Biosynex CryptoPS T1-band predicted 55.5% (5/9) and the T2-band 100% (5/5).

## Discussion

In Cameroon where 4.3% (Group Technique Central du Comite National de Lutte contre le VIH/SIDA, 2015) of adults are HIV-infected, we found in those presenting with <100 CD_4_ cells/ml, that 7.5% were serum CrAg positive using the IMMY LFA (5.9 and 4.3% using Biosynex CryptoPS and EIA, respectively) indicating a high risk of CM warranting implementation of systematic pre-ART CrAg screening in this population (World Health Organization, 2011). Nevertheless, doing that in a local context where current application of the HIV “test-and-treat” strategy (World Health Organization, 2016) does not recommend baseline pre-ART CD_4_ T-cell count remains challenging.

Depending on the test used for screening, one out of two to three patients with positive serum CrAg had asymptomatic CM, a finding like in other cohorts in the southern part of Africa (Mfinanga et al., 2015; Longley et al., 2016), suggesting that systematic LPs should be performed for all asymptomatic CrAg positive patients as a strategy to timely diagnose CM. Though we had high LP acceptability within the study, it is not always the case in routine settings with similar CM risks (Mfinanga et al., 2015; Longley et al., 2016), thus underlining the need for more acceptable methods to timely diagnose CM (Jackson and van der Horst, 2016). This could be achieved by offering LPs only to those with higher CrAg titres, as high blood titres are associated with presence of CM (Longley et al., 2016). Identifying those with high titres could be done either through serial dilutions of CrAg positive samples (Rajasingham and Boulware, 2015; Longley et al., 2016) or through the availability of a CrAg test capable of directly identifying those with high titres (Jackson and van der Horst, 2016; Sriruttan et al., 2016). Serial dilution is expensive, technically demanding, particularly for low-resource areas, requires a laboratory setting and prolongs diagnostic time. In our study, the Biosynex CryptoPS semi-quantitative T2-band, identified patients with high titres and these high titres correlated with laboratory evidence of asymptomatic CM (Longley et al., 2016). Such promising performance could be invaluable in CM diagnostic algorithms especially in settings with low LP uptake.

During the follow up period, there was no incident case of CM, a finding comparable to that of a South African cohort (Longley et al., 2016) where pre-emptive treatment was administered at the same tapering doses (World Health Organization, 2011). In rural Uganda, using 800 mg/day preemptively for only 4 weeks (Pac et al., 2015) yielded similar results within 6 months of follow up. However, another recent Ugandan cohort described a failure of fluconazole administered at this recommended pre-emptive dose in preventing incident CM (HR: 5.4) and death (HR: 3.2) within 6 months of screening and this failure was attributed to baseline CrAg titres ≥1:160 (Morawski et al., 2016). Here, EIA titres of 1:160 was associated with CM, thereby suggesting that Ugandan patients at baseline might have had undiagnosed CM for which fluconazole at 800 mg/day is not suitable. Indeed, this dosage is not recommended for CM induction treatment (Rothe et al., 2013) but is more appropriate as pre-emptive therapy. Overall, these data iterate the importance of considering antigen titre measurement as a proxy to timely CM diagnosis.

Overall mortality during first year of ART, though high, was comparable to findings in other cohorts in resource limited setting (Lawn et al., 2008). We found some evidence of CrAg positivity and risk of death but not as strong as elsewhere (Liechty et al., 2007; Jarvis et al., 2009; Pac et al., 2015; Longley et al., 2016), and this association disappeared upon adjusting with other factors (Kapoor et al., 2015). Arguably, it could be due to our relatively smaller sample, but a plausible explanation could also be our approach of active CM case finding at baseline such that over time, risks of CM was similar in those found CrAg positive or negative. Notwithstanding, though mortality in CrAg negatives in our cohort was still unacceptably high, there were more early deaths in those screened as CrAg positive but only one was attributed to CM. CrAg positive patients may have other poorly understood factors that lead to death due to non-CM causes; or deaths may have been with undetected cryptococcosis. The precise cause of death is extremely hard to ascertain in this patient population.

Our study has some limitations. The number of patients screened for CrAg was lower than initially planned due to modifications in local guidelines on diagnosis and treatment of HIV which reduced the number of potentially eligible patients.

Nevertheless, we believe that our findings may facilitate management strategies of cryptococcosis in Sub-Saharan African countries based on excellent performances of both POC tests used. Considering EIA as the reference standard, both POC had excellent sensitivity while specificity was slightly better in serum for Biosynex CryptoPS. Interestingly, though urine screening provided false positive results with the LFA IMMY as previously reported (Longley et al., 2016; Brito-Santos et al., 2017), Biosynex CryptoPS was not associated with false positives in urine.

Acknowledging the constraints around serial dilution for titre determination and considering that Biosynex CryptoPS T2-band directly uncovers those with high titres and CM, points out urgent broad evaluation of this new test as a timely tool for asymptomatic CM diagnosis.

## Conclusion

The prevalence of CrAg and asymptomatic CM in Cameroon is unexpectedly high, thereby suggesting that CM burden may also be comparably high as described in the Southern part of Africa. There is urgent need to integrate as part of HIV comprehensive care package, systematic CrAg screening and pre-emptive fluconazole therapy as a strategy to reduce HIV-associated CM morbidity and mortality. The Biosynex CryptoPS test is comparable to the IMMY LFA as POC CrAg screening test with apparently less false positive results in urine. The T2-band of Biosynex CryptoPS, provides a potentially useful surrogate diagnosis of CM that should prompt lumbar puncture and/or CM induction antifungal therapy.

## Author Contributions

ET, TH, OL designed the study. ET, CK, ED, SK-S, LM, and MF significantly contributed in data acquisition. ET, OL, and FD analyzed the data and together with TH, AL and SM, they did data interpretation. ET and OL drafted the manuscript. TH, AL, SM, FD, ET, OL, SK-S, and ED revised the manuscript. All authors approved the final manuscript and agreed to be accountable for all aspects of the work.

## Conflict of Interest Statement

FD was involved in the development of the Biosynex CryptoPS test. The other authors declare that the research was conducted in the absence of any commercial or financial relationships that could be construed as a potential conflict of interest.
